# Smart scaffolds in tissue regeneration

**DOI:** 10.1093/rb/rby007

**Published:** 2018-04-17

**Authors:** Samad Ahadian, Ali Khademhosseini

**Affiliations:** 1 Toronto General Research Institute, University Health Network, Toronto, ON M5G 2C4, Canada; 2 Department of Bioengineering; 3 Department of Radiology; 4 Department of Chemical and Biomolecular Engineering; 5 Center for Minimally Invasive Therapeutics (C-MIT); 6 California NanoSystems Institute (CNSI), University of California, Los Angeles, CA 90095-1600, USA

**Keywords:** drug delivery, scaffolds, regenerative mechanism

## Abstract

Recent advances in biofabrication technologies and chemical synthesis approaches have enabled the fabrication of smart scaffolds that aim to mimic the dynamic nature of the native extracellular matrix. These scaffolds have paved the way for tissue regeneration in a dynamic and controllable manner.

Natural tissues contain cells and growth factors embedded in spatially defined and heterogeneous extracellular matrix (ECM). The complex nature of the ECM is attributed to the nanoscale arrangement of molecules in the ECM that form fibrils at the microscale and directionally align fibers at the macroscale. The ECM controls not only tissue’s mechanical and biological properties, but also the dynamics of cells, soluble factors, nutrients and waste products within tissues.

Since the implantation of stainless steel as the first artificial hip in 1929, the path was opened to design and use biomaterials as artificial body parts. Further advances in materials science and engineering led to the discovery of temporary implants that would be able to restore tissue loss followed by biodegradation in the body. This shift caused the development of a variety of tissue scaffolds. Later, it was found that mechanical properties of scaffolds should be also compatible to the host tissue’s mechanics. An important and yet challenging design requirement for scaffolds is recapitulating the dynamic nature of the ECM. Smart scaffolds have recently emerged to fulfill this requirement by providing bio-responsive and structurally tunable scaffolds. These scaffolds could deliver biomolecules and release them in a precise and programmable manner. Interestingly, some smart scaffolds are able to modulate the host tissue response and further increase the therapeutic efficiency of scaffolds *in vivo*. Here, we provide a brief commentary on smart scaffolds in tissue regeneration from their advanced fabrication strategies to their performance *in vivo*.

## Biofabrication of smart scaffolds

**Figure 1:**
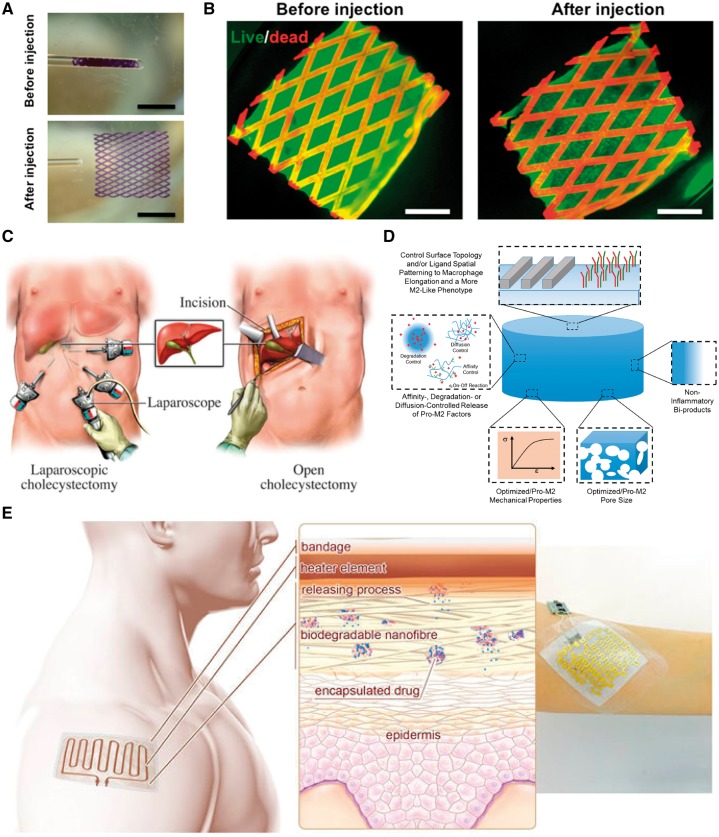
Smart scaffolds in tissue regeneration. **(A)** Pictures of shape-memory scaffold before and after the injection through the glass tube. Scale bars, 2.5 mm. Adapted with permission from ref. [1]. **(B)** Fluorescence pictures of live and dead rat cardiomyocytes (green and red colors, respectively) on the shape-memory scaffold before and after the injection. The scaffold showed autofluorescence in the red channel. Scale bars, 1 mm. Adapted with permission from ref. [1]. **(C)** Schematic of implantation of an engineered tissue on shape-memory scaffold in a minimally invasive manner using laparoscopic surgery compared with the tissue implantation using conventional surgery. **(D)** Characteristics of an ideal smart scaffold to modulate the inflammatory response of the body. Adapted with permission from ref. [17]. **(E)** schematic of a smart delivery system equipped with electronics and heater to release drugs embedded in nanofiber scaffolds. Adapted with permission from ref. [15]

The architecture of tissue scaffolds is of great importance. The porosity and interconnected network of scaffolds ensure cell penetration and diffusion of nutrients and waste products within tissues. Microfabrication techniques have enabled us to make versatile scaffold architectures for different tissue engineering applications. However, only specific architectures would result in smart scaffolds with the controllable structure. For example, we recently used a micromolding technique to make a lattice-shaped elastomeric scaffold with shape-memory property [1]. The folded scaffold in a small-diameter tube could memorize its physical structure and recover to it upon the injection (Fig. 1A). Interestingly, viability and function of engineered tissues on the scaffold were not affected via the injection (Fig. 1B). This smart scaffold has opened a new path for delivery of engineered tissues in the body in a minimally invasive manner (Fig. 1C). Other scaffold materials could possess this shape-memory property providing that they are soft, and relatively pliable and tough. Carbon nanotubes [2] and graphene [3] can also be integrated with shape-memory scaffolds as flexible electrodes to further enhance the functionality of scaffolds in tissue stimulation and sensing *in vivo*. Shape-memory scaffolds are not limited to mechanically flexible scaffolds with specific designs. Other external stimuli, such as electricity, heat and solubility can trigger scaffolds to memorize their permanent shape through a transient phase [4]. However, biocompatibility of shape-memory process and its relevance to *in vivo* and clinical conditions should be further investigated.

Primitive cells in a sheet-like arrangement go through morphogenetic processes to shape 3D tissues in the body. Origami-based smart scaffolds have been inspired by this principle to fabricate complex tissue constructs [5]. The fabrication process is rather simple and relies on internal stresses within scaffolds to transform scaffold sheets into 3D scaffold structures. Computer-aided designs have enhanced the complexity and robustness of origami-based scaffolds. Hydrogels are ideal materials to make origami-based smart scaffolds as their degree of swelling can generate internal stress in the materials [6]. However, the reversibility of swollen origami-based hydrogels still remains as a challenge.

Four-dimensional printing is a novel technology to fabricate 3D smart scaffolds with programmable shape change over time [7]. This technology aims to mimic the dynamic and complex architecture of the ECM. In a breakthrough work, Gladman *et al.* [8] used a biomimetic hydrogel capable of 4D printing of target micropatterned shapes. The printed architectures changed their shapes by immersing in water resulting in complex 3D morphologies. Prior to using this hydrogel as a smart scaffold for tissue regeneration, one should ensure cell viability and tissue functionality on the printed scaffolds. The ability of photolabile gelatin methacryloyl hydrogel in direct printing of cell-laden hydrogels [9] is an asset for 4D bioprinting technology to fabricate tissue constructs in macroscale. These works have paved the way for fabrication of bioprinted and smart tissue constructs that could temporally evolve their structure during the tissue morphogenesis. To this end, specific bioinks should be designed to meet the structural heterogeneity and functionality of different tissues in the body.

## Performance of smart scaffolds *in vivo*

Historically, minimizing inflammatory response of the body to engineered tissue constructs was desirable for successful tissue implantation. More recently, the macrophage invasion of tissue constructs as the natural immune defense has been manipulated to increase the healing efficiency of implanted tissues. Smart scaffolds can be designed to dictate favorable immune response of the body toward implanted tissues [10]. For instance, we showed that hydrogel micropatterns significantly affect the macrophage polarization from pro-inflammatory to anti-inflammatory responses [11]. Temperature-responsive microgrooves could also be ideal platforms to engineer the macrophage polarization [12]. In general, some topography cues and soluble factors (e.g. tumor necrosis factor-α, interleukin-1β and interleukin-6) can mediate the host tissue response. Therefore, smart scaffolds can be designed to provide such biophysical and biochemical cues and serves as novel immuno-informed biomaterials (Fig. 1D). Such biomaterials would be also able to recruit natural stimuli in the site of injuries (e.g. hypoxia and endogenous mesenchymal stem cells) for the polarization of macrophages to anti-inflammatory ones and thereby enhance the tissue healing and regeneration.

Smart scaffolds stand as delivery vehicles for controlled release of different biomolecules. The delivery could be triggered using external stimuli (e.g. pH, temperature and light) or could be done simultaneously as a result of programmable biodegradation of scaffolds [13]. In a recent work, Culver *et al.* [14] developed an analyte-responsive hydrogel for biosensing and drug delivery. This material is able to incorporate inherent molecular recognition of biomolecules in the hydrogel network and by this way increases the selectivity and accuracy of delivery process. In another study, we fabricated a biodegradable scaffold for temporal release of drugs with heat (Fig. 1E) [15]. This scaffold has a potential for electronically controlled release of drugs in wound dressings or surgical meshes. Advanced smart scaffolds used as delivery systems would be able to specifically interact with target tissues or organs in the body and release their contents with required release kinetics as a response to signals from target cells or local ECM. With the advent of new therapeutic agents, it is necessary to develop more smart scaffolds to deliver such agents in an efficient and controllable manner.

## Conclusion and future perspectives

There has been great progress in synthesis and biofabrication of smart scaffolds in tissue regeneration. Current synthesis approaches in terms of self- or forced-assemblies allow the incorporation of bio-recognition moieties or biomolecules (e.g. proteins, growth factors and peptides) into the molecular structure of scaffolds. Different functional monomers, oligomers or macromolecules have been combined with biological segments. From a microscale point of view, such hybrid and bio-responsive materials are able to interact with cells and mediate cell-cell communication in biological environments. However, biofabrication of such scaffold structures need further investigation to obtain 3D smart scaffolds in a scale-up, cost-effective, and reproducible manner. More importantly, the fabricated scaffolds should precisely represent the hierarchical sequences and functionality of biological components in the scaffolds. Therefore, the biofabrication process would preserve the bio-responsiveness and dynamics of smart scaffolds. Since these smart and functional scaffolds aim to mimic the multifunctionality of natural ECM, ongoing research should also focus on biofabrication of multi-faced smart scaffolds (e.g. 4D printed scaffolds incorporating growth factors to mediate the natural inflammatory response).

The European technology platform declared that smart biomaterials could play a major role in enabling technologies in human tissue regeneration [16]. Although significant progress has been made in understanding chemical, physical and biological properties of smart scaffolds, few scaffolds have met the demands for pre-clinical or clinical applications. In particular, long-term stability and performance, integration to native tissues, ability to control in deep tissues, and potential side effects of smart scaffolds should be further studied. The development of shape-memory scaffolds in minimally invasive surgical procedures is critically important. Such scaffolds have already shown a promising way for the implantation of engineered tissues in the body avoiding open surgery and post-surgery complications. However, the off-the-shelf availability and functionality of scaffolds should be assessed from a clinical perspective.

In summary, we hope that smart scaffolds would find their important role in fabricating functional tissue constructs. These scaffolds may offer on-demand and controllable biomolecule delivery to target tissues and organs in the body. In addition, application of smart scaffolds in biosensing, biorobotics, and imaging could give us remarkable insights for *in situ* monitoring and controlling the biological response of scaffolds *in vivo*.

*Conflict of interest statement*. None declared.
